# Monoacylglycerol Lipases Act as Evolutionarily Conserved Regulators of Non-oxidative Ethanol Metabolism*

**DOI:** 10.1074/jbc.M115.705541

**Published:** 2016-03-31

**Authors:** Christoph Heier, Ulrike Taschler, Maja Radulovic, Philip Aschauer, Thomas O. Eichmann, Susanne Grond, Heimo Wolinski, Monika Oberer, Rudolf Zechner, Sepp D. Kohlwein, Robert Zimmermann

**Affiliations:** From the ‡ Institute of Molecular Biosciences, University of Graz and; § BioTechMed-Graz, 8010 Graz, Austria

**Keywords:** fatty acid metabolism, lipid droplet, lipid metabolism, Saccharomyces cerevisiae, serine esterase, ethanol metabolism, monoacylglycerol lipase

## Abstract

Fatty acid ethyl esters (FAEEs) are non-oxidative metabolites of ethanol that accumulate in human tissues upon ethanol intake. Although FAEEs are considered as toxic metabolites causing cellular dysfunction and tissue damage, the enzymology of FAEE metabolism remains poorly understood. In this study, we used a biochemical screen in *Saccharomyces cerevisiae* to identify and characterize putative hydrolases involved in FAEE catabolism. We found that Yju3p, the functional orthologue of mammalian monoacylglycerol lipase (MGL), contributes >90% of cellular FAEE hydrolase activity, and its loss leads to the accumulation of FAEE. Heterologous expression of mammalian MGL in *yju3*Δ mutants restored cellular FAEE hydrolase activity and FAEE catabolism. Moreover, overexpression or pharmacological inhibition of MGL in mouse AML-12 hepatocytes decreased or increased FAEE levels, respectively. FAEEs were transiently incorporated into lipid droplets (LDs) and both Yju3p and MGL co-localized with these organelles. We conclude that the storage of FAEE in inert LDs and their mobilization by LD-resident FAEE hydrolases facilitate a controlled metabolism of these potentially toxic lipid metabolites.

## Introduction

The consumption of ethanol has a widespread social tradition among many populations worldwide. Although moderate ethanol intake has been regarded as beneficial to cardiovascular health, chronic alcohol abuse is associated with an increased risk of pancreatitis, cardiomyopathy, liver disease, and cancer (1–6). Numerous studies suggest that the metabolic conversion of ethanol and the resulting generation of toxic metabolites play a central role in the development of alcohol-related diseases (7).

Eukaryotic cells metabolize ethanol via oxidative and non-oxidative pathways. In humans, >90% of the ingested ethanol is removed by the liver via sequential oxidation to acetaldehyde and acetate (7). In addition to oxidative pathways, ethanol can be metabolized by several non-oxidative pathways that yield phosphatidylethanol, ethyl sulfatides, ethyl glucuronides, and fatty acid ethyl esters (FAEE),^3^ respectively (7–9). In humans, the quantitative contribution of non-oxidative pathways to ethanol metabolism is minor. However, it has been suggested that chronic accumulation of specific non-oxidative ethanol metabolites such as FAEE contributes to ethanol toxicity (10, 11). This notion is supported by several *in vitro* and *in vivo* studies that demonstrate toxicity for FAEE to cultured cells and tissues (12–16).

FAEE are formed either via condensation of ethanol with fatty acids (FA) catalyzed by FAEE synthases or by transesterification of acyl-CoA with ethanol by acyl-CoA ethanol *O*-acyltransferases (AEAT). FAEE synthase and AEAT activities have been described in a multitude of tissues including liver, pancreas, heart, and brain (10, 11, 17–21). Although the molecular identity of enzymes responsible for FAEE synthesis *in vivo* is not known, biochemical evidence suggests the involvement of carboxylesterases and/or lipases (22–25). FAEE may be hydrolyzed to ethanol and FAs by FAEE hydrolases (19, 21, 26). To date, however, little is known about FAEE catabolism as well as enzymes and their regulators governing this process.

FAEE are synthesized in ethanol producing *Saccharomyces cerevisiae* (baker's yeast), making it an attractive model to study the enzymology of FAEE metabolism (27–30). We hypothesized that conserved enzymatic pathways may exist in yeast and mammalian cells that contribute to FAEE metabolism. Using a biochemical screening approach, we indeed identified monoacylglycerol lipases from yeast and mammals as evolutionarily conserved FAEE hydrolases that impact non-oxidative ethanol metabolism.

## Experimental Procedures

### 

#### 

##### Materials

Ethyl palmitate, ethyl palmitoleate, ethyl stearate, ethyl oleate, methyl nonadecanoate, 1-oleoyl-*rac*-glycerol, trioleoylglycerol, Orlistat (tetrahydrolipstatin), and phenylmethylsulfonyl fluoride (PMSF) were obtained from Sigma. Cholesteryl oleate was obtained from Avanti Polar Lipids (Alabaster, AL). Palmitic acid (9,10-^3^H(N)) and 1-palmitoyl *rac*-[1-^14^C]glycerol were obtained from American Radiolabeled Chemicals, Inc. (St. Louis, MO). Palmitic acid (1-^14^C) and oleic acid (1-^14^C) were obtained from Moravek Biochemicals (Brea, CA). Monoacylglycerol (MAG) lipase inhibitor JZL-184 was obtained from Abcam (Cambridge, UK).

##### Strains and Media

The yeast strains BY4742 (*MATa his3*Δ*1 leu2*Δ*0 lys2*Δ*0 ura3*Δ*0*), *yju3*Δ *(MATa his3*Δ*1 leu2*Δ*0 lys2*Δ*0 ura3*Δ*0 ykl094w*::*kanMX4*), and *vma11*Δ (*MATa his3*Δ*1 leu2*Δ*0 lys2*Δ*0 ura3*Δ*0 ypl234c*::*kanMX4*) were obtained from Euroscarf (Frankfurt, Germany). The strain Yju3-GFP (*MAT*a *YKL094w-GFP-HIS3MX6 leu2*Δ*0 met15*Δ*0 ura3*Δ*0*) was obtained from Invitrogen. The strain Elo3-RFP (*MATa YLR372w-RFP-kanMX4 his3*Δ*1 leu2*Δ*0 lys2*Δ*0 ura3*Δ*0*) was generated by homologous recombination of a mRFP-*kan*MX4 cassette at the 3′-site of the *ELO3* open reading frame. The strain Yju3-GFP/Elo3-RFP (*MAT*a *YJU3-GFP*::*HIS3MX6 ELO3-RFP*::*kanMX4 his3*Δ*1 leu2*Δ*0 met15*Δ*0 ura3*Δ*0*) was generated by standard genetic crosses and tetrad dissection as described previously (31). Yeast cells were grown in YPD medium containing 1% yeast extract, 2% peptone, and 2% glucose or in synthetic minimal medium (MM) containing 0.67% yeast nitrogen base, 2% glucose, or in YPE media containing 4% ethanol instead of glucose, and the respective amino acids and nucleobases. Yeast transformants carrying expression plasmids were selected in uracil-free medium. Geneticin resistance was scored on YPD plates containing 200 mg/liter Geneticin (G418; Calbiochem, Merck, Whitehouse Station, NJ). Yeast strains were grown at 30 °C on a rotary shaker with vigorous aeration. Cell growth was monitored with a Casy® TTC cell counter (Schärfe System, Reutlingen, Germany) or by measuring the optical density at 600 nm (*A*_600_).

##### Cloning of Recombinant Proteins

The open reading frames *YKL140w*/*TGL1*, *YMR313c*/*TGL3*, *YKR089c*/*TGL4, YOR081c*/*TGL5*, *YBR204c*/*LDH1*, *YOR059c*/*LPL1*, *YBR177c*/*EHT1*, *YPR147c*, *YGL144c*/*ROG1*, and *YKL094w*/*YJU3* were amplified by PCR using genomic DNA obtained from BY4742 as template and the following primers: TGL1_fw, 5′-GAT CGG ATC CAT GTA CTT CCC CTT TTT AGG-3′; TGL1_rv, 5′-GAT CGT CGA CTT CTT TAT TTA GAG CAT CCA-3′; TGL3_fw, 5′-GAT CAC TAG TAT GAA GGA AAC GGC GCA GG-3′; TGL3_rv, 5′-GAA TGT CGA CCC TAC TCC GTC TTG CTC TTA-3′; TGL4_fw, 5′-GAA TAC TAG TAT GAG CAG CAA AAT ATC AG-3′; TGL4_rv, 5′-GAA TGT CGA CTT GAG TAA AAC TGG TGG TGC-3′; TGL5_fw, 5′-GAT CGG ATC CAT GTC TAA TAC CTT GCC AG-3′; TGL5_rv, 5′-GAA TAT CGA TAT TTT GAA AAA TGT CTG AAT-3′; LDH1_fw, 5′-GAT CAC TAG TAT GAA TAT GGC AGA ACG TG-3′; LDH1_rv, 5′-GAA TGT CGA CCA ATT TGG AAT TAT CAA TC-3′; YOR059c_fw, 5′-GAA TGG ATC CAT GAC TTC GGA TAA ACA CC-3′; YOR059c_rv, 5′-GAA TAT CGA TAT TAC TCT TGT GCA TCA AG-3′; EHT1_fw, 5′-GAT CAC TAG TAT GTC AGA AGT TTC CAA ATG-3′; EHT1_rv: 5′-GAT CGT CGA CTA CGA CTA ATT CAT CAA ACT-3′; YJU3_fw, 5′-GAT CAC TAG TAT GGC TCC GTA TCC ATA CAA-3′; YJU3_rv, 5′-GAT CGT CGA CTG GTT TAG CTT CGG TCG TGG-3′; ROG1_fw; 5′-AAC TCC CGG GAT GTC TCT GAC ACC AAC TA-3′; ROG1_rv; 5′-AAC TGA ATT CTT GTA CCA AAT CAC TAT TCA-3′.

PCR products were purified by agarose gel electrophoresis and were inserted into the multiple cloning site of the pUG35 vector using the restriction sites for SpeI, SalI, BamHI, XmaI, EcoRI, or ClaI to create C-terminal fusions with GFP (32). The construction of a pUG36-derived plasmid encoding GFP-tagged murine MGL and of a pcDNA4/Hismax C-derived plasmid encoding His_6_-tagged mouse MGL was described previously (31). For the generation of a lentivirus encoding ECFP-tagged murine MGL, the respective coding sequence was amplified by PCR from the pcDNA4/Hismax C construct using the following primers: MGL_fw, 5′-GAT CCT CGA GGC CAC CAT GCC TGA GGC AAG TTC AC-3′; MGL_rv, GAA TCC GCG GGG GTG GAC ACC CAG CTC CTG. The PCR product was subcloned into a pECFP-N1 vector (Clontech, Mountain View, CA) using the restriction sites for XhoI and SacII. The resulting plasmid was digested with XhoI and NotI, and the fragment encoding for a MGL-ECFP fusion protein was ligated into the pLVX IRES Puro vector (Clontech). Lentivirus encoding for ECFP was constructed by digesting pECFP-N1 with XhoI and NotI and by ligating the resulting fragment into the pLVX IRES Puro vector.

##### Expression of Recombinant Proteins in Yeast and Preparation of Cell Extracts

Plasmids were transformed into yeast using the lithium acetate method (31). The *CEN* plasmids pUG35 and pUG36 contain the methionine-regulated *MET25* promotor (32); for the expression of GFP-tagged recombinant proteins transformants were cultivated in MM medium lacking uracil and methionine. Cells were harvested in the late logarithmic (log) phase and were disrupted with glass beads in a buffer containing 0.25 m sucrose, 1 mm EDTA, 1 mm DTT, 2 mg/liter antipain, 1 mg/liter pepstatin, and 20 mg/liter leupeptin (buffer A) using a Merckenschlager homogenizer (Braun Biotech International GmbH, Melsungen, Germany). Cell debris and nuclei were removed by centrifugation at 1000 × *g* and 4 °C for 10 min, and the cleared homogenates were used for further experiments.

##### Subcellular Fractionation

1000 × *g* supernatants of yeast cell homogenates were overlaid with 50 mm potassium phosphate buffer, pH 7.5, containing 100 mm KCl, 1 mm EDTA, 2 mg/liter antipain, 1 mg/liter pepstatin, and 20 mg/liter leupeptin and centrifuged at 100,000 × *g* and 4 °C for 1 h. Floating lipid droplets (LDs), cytosolic fractions, and membrane fractions were collected and resuspended in buffer A. For protein determination, aliquots of the cell fractions were mixed with a 4-fold volume of acetone and incubated overnight at −20 °C to precipitate proteins and to remove lipids. After centrifugation at 10,000 × *g* for 30 min, precipitated protein was dissolved in 0.1% SDS containing 0.3 m NaOH, and protein concentration was determined with a BCA protein assay according to the manufacturer's instructions (Pierce® BCA^TM^ Protein Assay kit, Thermo Scientific, Waltham, MA) using bovine serum albumin (BSA) as a standard.

##### Purification of Yju3p

Purification of the His_6_-tagged Yju3p from *Escherichia coli* was performed via immobilized metal ion affinity chromatography as described previously (31, 33).

##### Growth Tests

Yeast strains were cultivated overnight at 30 °C in liquid YPD or MM medium. 10 *A*_600_ units were harvested, serially diluted (1:10), and applied onto agar plates containing YPE, YPD, or ΜΜ (as indicated) with increasing concentrations of ethanol. Growth was monitored after 2 days at 30 °C.

##### Generation of AML-12 Cells Stably Overexpressing ECFP and MGL_ECFP and Preparation of Cell Homogenates

AML-12 hepatocytes (ATCC #CRL-2254) were maintained in a 1:1 mixture of Dulbecco's modified Eagle's medium and Ham's F-12 medium (Gibco) supplemented with 5 μg/ml insulin, 5 μg/ml transferrin, 5 ng/ml selenium, 40 ng/ml dexamethasone, 10% fetal bovine serum, 100 IU/ml penicillin, and 100 μg/ml streptomycin at 37 °C, 5% CO_2_, and 95% humidity. Lentiviral particles harboring pLVX IRES Puro vectors encoding for ECFP or ECFP-MGL, respectively, were generated in Hek293T cells according to the manufacturer's instructions (Clontech). Before transduction AML-12 cells were seeded into 6-well plates at a density of 300,000 cells per well. Cells were incubated for 24 h with lentivirus-containing supernatants in the presence of 8 μg/ml Polybrene. To select for stable expression, cells were maintained for 7 days in medium containing 1.5 μg/ml puromycin.

##### Preparation of AML-12 Cell Homogenates and Subcellular Fractionation

For the preparation of homogenates, cells were washed with phosphate-buffered saline (PBS), collected using a cell scraper, and disrupted in buffer A by sonication (Virsonic 475; Virtis, Gardiner, NJ). Nuclei and unbroken cells were removed by centrifugation (1000 × *g*, 4 °C, for 10 min). For the isolation of LDs, cell homogenates were prepared by nitrogen cavitation in buffer A containing 100 μmol/liter PMSF and 1 μmol/liter JZL-184. Cell debris was removed by centrifugation (1000 × *g*, 4 °C, for 10 min), and the resulting homogenate was overlaid with PBS containing 100 μmol/liter PMSF and 1 μmol/liter JZL-184. After centrifugation at 100,000 × *g* for 1 h, floating LDs, cytosol, and membrane fractions were individually recovered.

##### Preparation of Radiolabeled Ethyl Palmitate

^3^H-Labeled palmitic acid (1 mCi in 100 μl of ethanol) was mixed with toluene (500 μl) in the presence of 100 μg of butylated hydroxytoluene. After the addition of 3 ml of 2% HCl in ethanol, the reaction mixture was vortexed and incubated for 1 h at 100 °C. After cooling, ice-cold distilled H_2_O was added, and [^3^H]ethyl palmitate was extracted twice using hexane/chloroform (4:1, v/v) and brought to dryness. Samples were dissolved in chloroform/methanol (2:1, v/v) and further purified by solid phase extraction using a NH_2_ column (Stratagene, Santa Clara, CA).

##### Lipid Hydrolase Assays

MAG or FAEEs were emulsified by sonication in 200 mm Bistris propane buffer, pH 7, containing 10 mm CHAPS, 1 mm EDTA, and ^3^H-labeled tracers at a final specific activity of 2 μCi/ml. The final substrate concentration was 2 mm unless otherwise indicated. Samples were diluted in buffer A to yield protein concentrations of 0.2–1.2 mg/ml (yeast homogenates, cytosol, membranes), 0.01–0.1 mg/ml (yeast LDs), 0.2 μg/ml (purified Yju3p), or 1.0 mg/ml (AML-12 cell homogenates). Inhibitors were dissolved in DMSO and were incubated with samples at a final concentration of 1 μm at 37 °C for 15 min before the addition of the substrate. Assays were started by the addition of 100 μl of substrate to 100-μl samples in buffer A and incubated at 37 °C in a shaking water bath for 30 min unless otherwise indicated. Assays were stopped by the addition of 3.3 ml of methanol/chloroform/heptane (10:9:7, v/v/v) and 1 ml of 100 mm potassium carbonate/boric acid, pH 10.5. FAs were extracted into the upper phase by vortexing, and phase separation was achieved by centrifugation. The radioactivity in the upper phase was determined by liquid scintillation counting. Alternatively, substrates were prepared without radiolabeled tracers, and the release of free FAs was determined using a commercially available kit (NEFA reagent, Wako, Neuss, Germany) according to the manufacturer's instructions. All assays were performed in triplicate. For the determination of pH optima, a buffer system containing succinic acid, sodium dihydrogen phosphate, and glycine was used.

##### Thin-layer Chromatography (TLC) of Yeast Lipids

For the extraction of lipids, yeast cells were dispersed in chloroform/methanol (2:1, v/v) and disrupted using glass beads as described (31). Extracts were applied onto Silica G plates and separated using toluene as solvent. Lipids were visualized by carbonization at 120 °C using a solution containing 10% H_3_PO_4_ and 10% CuSO_4_ and identified by co-migration of authentic standards.

##### GC/MS Analysis of FAEE

For the determination of FAEEs, yeast lipids were extracted and resolved by thin-layer chromatography as described above. The spots co-migrating with FAEE were scraped off and extracted twice with chloroform. FAEE species were resolved by GC/MS and quantified using calibration curves for each species. Methyl nonadecanoate served as an internal standard to monitor the recovery of FAEE throughout the procedure.

##### Radiolabeling Experiments

Yeast lipids were labeled by incubating cells with [^14^C]palmitic acid and extracted with chloroform/methanol (2:1, v/v) as described previously (31). AML-12 cells were seeded into 6-well plates at a density of 300,000 cells/well and incubated for 16 h in standard medium. Radiolabeling of lipids was performed by incubating the cells for 12 h in medium containing 100 μmol/liter [^14^C]oleic acid (complexed to BSA, specific activity: 1 mCi/mmol) in the absence or presence of 1 μm JZL-184. To induce formation of FAEE the medium was supplemented with 1% ethanol and exchanged with fresh medium every 4 h. Lipids were extracted twice with hexane/isopropyl alcohol (3:2, v/v). Lipid extracts were applied onto Silica G plates and separated using either toluene or chloroform/acetone/acetic acid (90:8:1, v/v/v) as solvent systems. Lipids were identified using authentic standards, and the co-migrating radioactivity was measured by liquid scintillation counting.

##### Fluorescence Microscopy

Yeast cells were immobilized on agar slides and imaged using a Leica SP5 confocal microscope equipped with a Leica HCX 63 × 1.4 NA objective. GFP was excited at 488 nm, and emission was detected between 500 and 530 nm. LD540 was a kind gift of Christoph Thiele (University of Bonn) and used for the detection of LDs as described previously (34). RFP and LD540 were excited at 563 nm, and the emission was detected between 580 and 630 nm. AML-12 cells were seeded onto 8-well chamber slides (Sarstedt, Nümbrecht, Germany) and incubated for 24 h in medium containing 100 μmol/liter oleic acid (complexed to BSA) to induce LD formation. Afterward, cells were fixed for 20 min in PBS containing 4% paraformaldehyde. For the detection of LDs, HCS LipidTOX^TM^ Deep Red was used according to the manufacturer's instructions (Thermo Fisher Scientific, Waltham, MA). Cells were imaged using a Leica SP5 confocal microscope equipped with a Leica HCX 63 × 1.4 NA objective. ECFP was excited at 458 nm, and emission was detected between 470 and 530 nm. HCS LipidTOX^TM^ Deep Red was excited at 633 nm, and emission was detected between 650 and 700 nm.

##### Determination of Protein Concentration

Protein concentrations of cell extracts were determined using the Bio-Rad Protein Assay Kit according to the manufacturer's instructions using BSA as the standard. The concentrations of purified proteins were obtained by the absorbance at 280 nm using the NanoDrop® ND-1000 Spectrophotometer (PEQLAB Biotechnologie GmbH, Erlangen, Germany). Alternatively, protein concentrations were determined using the Pierce® BCA Protein Assay kit and BSA as the standard according to the manufacturer's protocol (Pierce® BCA^TM^ Protein Assay kit, Thermo Scientific).

##### Statistical Analysis

All measurements were performed in triplicate. Data are presented as the means ± S.D. Statistical significance was determined by Student's unpaired *t* test. Group differences were considered statistically significant for *p* < 0.05 (*). *K_m_* and *V*_max_ were determined by nonlinear regression analysis using GraphPad Prism 5.0 (GraphPad Software, San Diego, CA).

## Results

### 

#### 

##### Characterization of FAEE Hydrolase Activity in Yeast Homogenates

**FIGURE 1.**
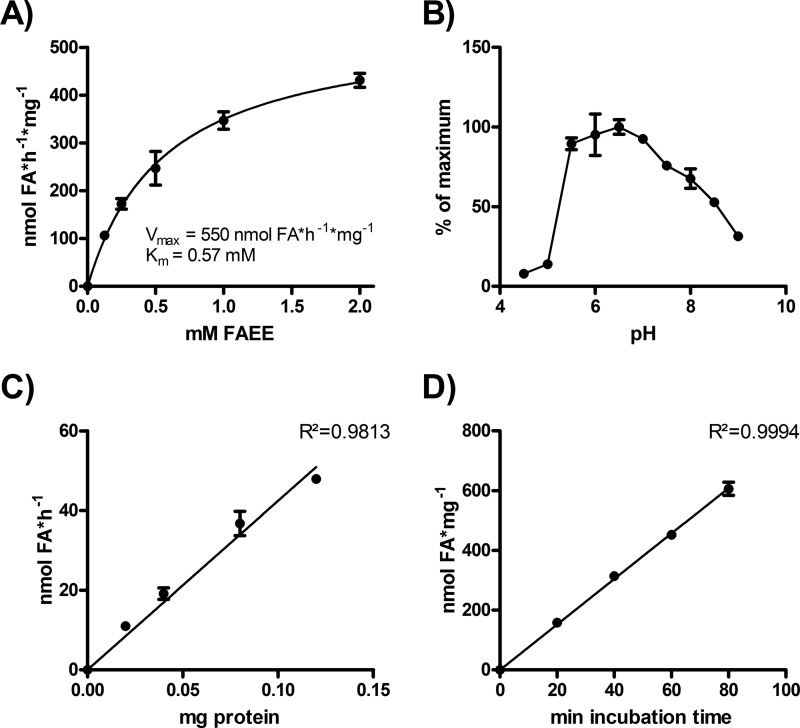
**Characterization of the endogenous FAEE hydrolase activity in yeast homogenates.** *A*, saturation kinetics of FAEE hydrolase activity. Samples were incubated with different concentrations of ethyl palmitate. *V*_max_ and *K_m_* were determined by nonlinear regression analysis. *B*, pH dependence of FAEE hydrolase activity. Samples were incubated in the presence of 2 mm ethyl palmitate at the indicated pH values. *C*, protein dependence of FAEE hydrolysis. Samples containing different amounts of protein were incubated with 2 mm ethyl palmitate. *D*, time dependence of FAEE hydrolysis. Samples were incubated with 2 mm ethyl palmitate. All assays were performed in triplicate using yeast 1000 × *g* supernatants. Data are representative of two independent experiments and are presented as the means ± S.D.

To identify FAEE hydrolases in yeast homogenates, we first optimized assay conditions using ethyl palmitate as a substrate. Endogenous FAEE hydrolase(s) present in total cell homogenates displayed an apparent *V*_max_ of 550 nmol × h^−1^ × mg^−1^ and a *K_m_* of 0.57 mm (Fig. 1*A*). The pH optimum of the reaction was between pH 6 and 7 (Fig. 1*B*). At pH 7, FAEE hydrolase activity showed a linear correlation with protein concentration (Fig. 1*C*), and the reaction was linear for up to 90 min (Fig. 1*D*).

##### Subcellular Distribution of FAEE Hydrolase Activity and Screening for Candidate Hydrolases

**FIGURE 2.**
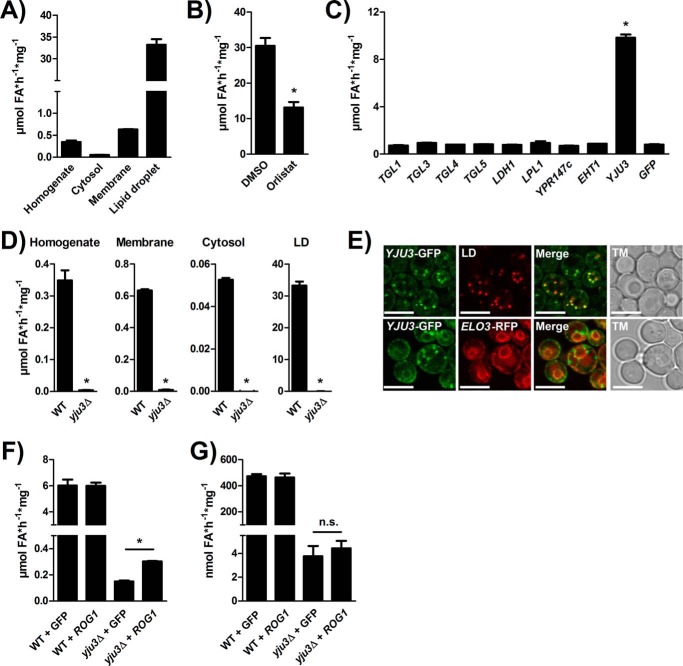
**Identification of Yju3p as FAEE hydrolase.** *A*, specific FAEE hydrolase activities of different subcellular fractions. Yeast homogenates were fractionated into cytosol, membranes, and LDs, and the specific FAEE hydrolase activity of each fraction was determined. *B*, LD-associated FAEE hydrolase activity in the absence and presence of 30 μm Orlistat. *C*, FAEE hydrolase activity of yeast homogenates overexpressing GFP-tagged serine hydrolases. *D*, FAEE hydrolase activity of WT and *yju3*Δ subcellular fractions. Yeast homogenates were fractionated into cytosol, membranes, and LDs, and the specific FAEE hydrolase activity of each fraction was determined. *E*, subcellular localization of Yju3p-GFP. Cells harboring a *YJU3*-GFP allele were imaged by confocal fluorescence microscopy. LD540 and Elo3p-RFP were used for the detection of LDs and the endoplasmic reticulum, respectively. Cells were cultivated in MM and imaged in the early log phase. *Scale bars*: 5 μm. *TM*, transmission. MAG hydrolase (*F*) and FAEE hydrolase (*G*) activity of WT and *yju3*Δ homogenates overexpressing *ROG1* or GFP. All assays were performed in triplicate and are representative of two independent experiments. Data are presented as the means ± S.D. Statistical significance was determined using Student's unpaired *t* test. *, *p* < 0.05; *n.s.*, not significant.

To determine the subcellular localization of FAEE hydrolase(s), we separated yeast homogenates into cytosol, membrane, and LD fractions and measured the specific activity in each fraction. The specific FAEE hydrolase activity was low in the cytosol, moderately increased in the membrane fraction, and substantially enriched (∼100-fold) in the LD fraction as compared with the homogenate (Fig. 2*A*). The serine hydrolase inhibitor Orlistat reduced the FAEE hydrolase activity of the LD fraction by 50% (Fig. 2*B*), suggesting the involvement of an LD-associated serine hydrolase(s) (35, 36). Yeast expresses several known LD-associated serine hydrolases with annotated functions in acylglycerol, sterol, and FAEE metabolism (28, 37–48). We thus overexpressed *YJU3, TGL1*, *TGL3*, *TGL4*, *TGL5*, *EHT1*, *LDH1*, *LPL1*, and *YPR147c* as GFP-tagged fusion proteins in wild-type yeast (WT) and performed FAEE hydrolase assays. Transformants overexpressing gene *YJU3*, encoding the major MAG lipase in yeast (31), displayed a 10-fold increase of the cellular FAEE hydrolase activity, whereas expression of the other open reading frames did not significantly change the cellular FAEE hydrolase activity compared with GFP-expressing control homogenates (Fig. 2*C*). Conversely, deletion of *YJU3* almost completely abolished FAEE hydrolase activity in cell homogenates as well as in isolated cytosolic, membrane, and LD fractions (Fig. 2*D*). Consistent with the subcellular distribution of activity, GFP-tagged Yju3p showed a dual localization on LDs and the endoplasmic reticulum, based on co-localization experiments with the LD-specific dye, LD540, and the endoplasmic reticulum resident Elo3p-RFP (Fig. 2*E*). To test if Rog1p, a second MAG lipase in yeast (49), harbors FAEE hydrolase activity we expressed the enzyme in WT and *yju3*Δ cells and performed hydrolase assays. *ROG1* expression moderately increased MAG hydrolase activity in *yju3*Δ but not in WT homogenates (Fig. 2*F*) confirming that the enzyme exhibits only minor activity compared with Yju3p; FAEE hydrolase activity of Rog1p was not detectable (Fig. 2*G*). Together, these data suggest that Yju3p, in addition to its role as a MAG lipase, also functions as the major FAEE hydrolase on LDs and cellular membranes. These observations further indicate that FAEE hydrolase activity is not a general feature of MAG hydrolases.

##### Purified Yju3p Exhibits FAEE Hydrolase Activity

**FIGURE 3.**
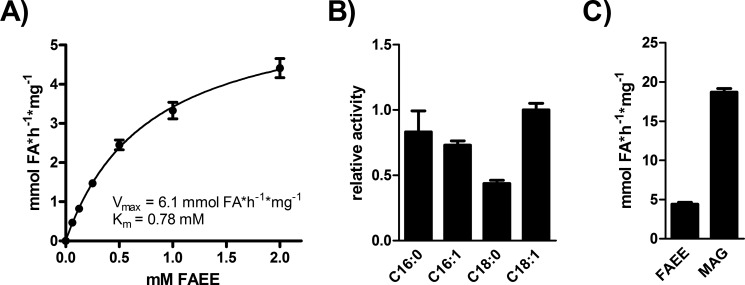
**FAEE hydrolase activity of purified Yju3p.** *A*, substrate saturation of purified Yju3p. Yju3p was incubated with substrates containing different concentrations of ethyl palmitate for 30 min. *B*, FAEE hydrolase activity of purified Yju3p against different FAEE species. Yju3p was incubated with 2 mm FAEE containing different acyl chains. The release of FA was determined by a coupled colorimetric assay. Data are presented as relative activities compared with C18:1 FAEE. *C*, comparison of FAEE and MAG hydrolysis catalyzed by purified Yju3p. Yju3p was incubated with 2 mm ethyl palmitate or palmitoylglycerol as substrate. *V*_max_ and *K_m_* were determined by nonlinear regression analysis. Assays were performed in triplicate and are representative of two independent experiments. Data are presented as the means ± S.D.

To confirm that Yju3p is a *bona fide* FAEE hydrolase we purified recombinant His_6_-tagged Yju3p from *E. coli* and determined its FAEE hydrolase activity *in vitro*. Yju3p exhibited a *V*_max_ of 6.1 mmol × h^−1^ × mg^−1^ protein and a *K_m_* of 0.78 mm against ethyl palmitate as substrate at neutral pH (Fig. 3*A*). As shown in Fig. 3*B*, Yju3p hydrolyzes ethyl palmitate (C16:0), ethyl palmitoleate (C16:1), and ethyl oleate (C18:1) with comparable efficiency, whereas ethyl stearate (C18:0) was hydrolyzed at an ∼2-fold lower rate as compared with the other molecular species. Yju3p was initially characterized as a MAG lipase; thus, we compared its activity against ethyl palmitate and palmitoylglycerol as substrates (31). Indeed, palmitoylglycerol was hydrolyzed at a 4-fold higher rate than ethyl palmitate under our experimental conditions (Fig. 3*C*), demonstrating that Yju3p prefers MAG but also robustly hydrolyzes FAEE *in vitro*.

##### Yju3p Controls Cellular FAEE Levels

**FIGURE 4.**
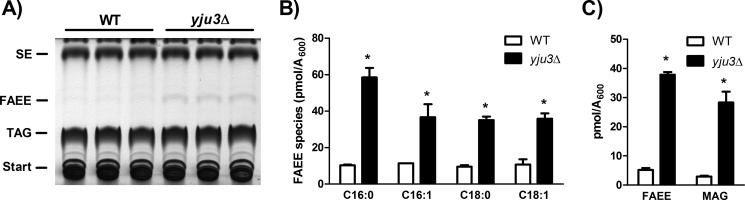
**Impact of *YJU3* deficiency on cellular FAEE metabolism.** *A*, lipid analysis of WT and *yju3*Δ. Cells were harvested in the late log-phase. Lipids were resolved by TLC and identified by co-migration of authentic lipid standards. *B*, levels of individual FAEE species in WT and *yju3*Δ cells. FAEE were isolated by TLC and quantified by GC/MS. *C*, incorporation of [^14^C]palmitic acid into MAG and FAEE in WT and *yju3*Δ cells. Cells cultivated to the late log phase were labeled with [^14^C]palmitic acid. Lipids were resolved by TLC, and radioactivity associated with MAG and FAEE was determined. Data are presented as the means ± S.D. (*n* = 3). Statistical significance was determined using Student's unpaired *t* test. *, *p* < 0.05.

To investigate if loss of Yju3p affects cellular FAEE metabolism, we analyzed cellular FAEE levels of logarithmically growing *yju3*Δ and WT cells. *YJU3* deficiency led to an accumulation of intracellular FAEE as compared with WT, whereas steryl ester (SE) and triacylglycerol (TAG) levels were not significantly affected (Fig. 4*A*). The major FAEE species detected in WT cells by GC/MS analysis contained C16:0, C16:1, C18:0, and C18:1 FAs, which were increased 3–6-fold in *yju3*Δ cells (Fig. 4*B*). We next investigated the effect of *YJU3* deficiency on the metabolism of FAEE and MAG by measuring the incorporation of radiolabeled FAs into both lipid classes. Loss of Yju3p resulted in a similar accumulation of radioactivity in both MAG and FAEE (Fig. 4*C*). Thus, Yju3p simultaneously controls cellular FAEE and MAG levels.

##### Loss of Yju3p Does Not Affect Ethanol Sensitivity

**FIGURE 5.**
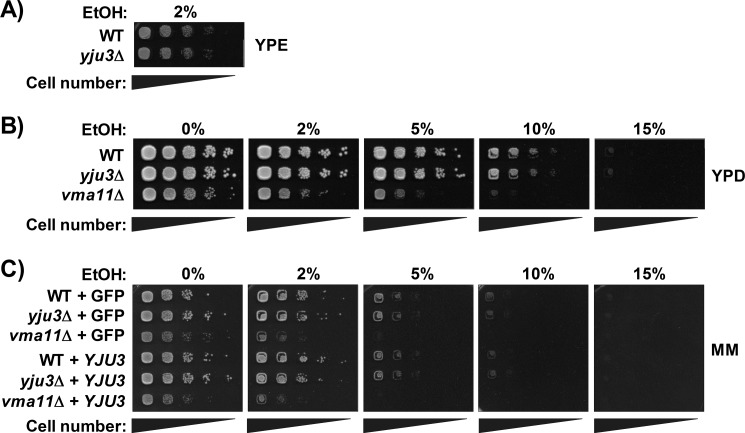
**Impact of *YJU3* deficiency or overexpression on ethanol sensitivity.** *A*, WT and *yju3*Δ were grown at 30 °C for 2 days on YPE. *B*, WT and *yju3*Δ were grown at 30 °C for 2 days on YPD in the absence or presence of increasing ethanol concentrations. *vma11*Δ was used as a negative control. *C*, WT, *yju3*Δ, and *vma11*Δ cells transformed with plasmids encoding GFP or *YJU3*-GFP were grown at 30 °C for 2 days on ΜM lacking uracil and methionine in the absence or presence of increasing ethanol concentrations. Growth tests are representative of two independent experiments.

To test whether impaired FAEE catabolism in *yju3*Δ cells affects growth on ethanol, we performed plate tests in the presence of different ethanol concentrations. As shown in Fig. 5, *yju3*Δ cells grew similar to WT on YPE or YPD and MM in the absence of ethanol. Growth on 2% ethanol as the sole carbon source was not impaired, indicating fully intact mitochondrial/respiratory function (Fig. 5*A*). In the presence of glucose, 2–15% ethanol progressively inhibited growth of WT and *yju3*Δ cells to the same extent (Fig. 5, *B* and *C*). As a negative control, a strain lacking the vacuolar ATPase Vma11p was used that exhibited impaired growth and increased ethanol sensitivity compared with WT on both YPD and MM (50, 51). Episomal overexpression of *YJU3* in WT, *yju3*Δ, or *vma11*Δ cells did not affect growth as compared with cells expressing GFP in the absence or presence of ethanol (Fig. 5*C*). Thus, loss or overexpression of Yju3p did not markedly affect cellular growth on standard laboratory media or ethanol.

##### The FAEE Hydrolase Activity of Yju3p Is Evolutionarily Conserved

**FIGURE 6.**
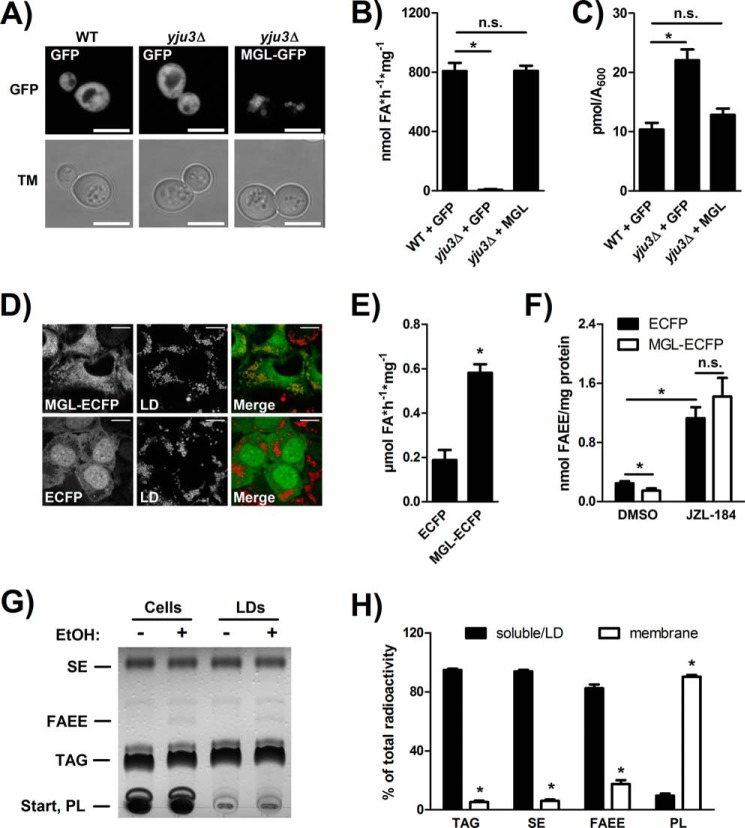
**Subcellular localization and FAEE hydrolase activity of murine MGL.** *A*, confocal fluorescence microscopy of WT yeast overexpressing GFP or GFP-MGL. Cells were cultivated in MM lacking uracil and methionine and imaged in the late log phase. *Scale bars*: 5 μm. *TM*, transmission. *B*, incorporation of [^14^C]palmitic acid into FAEE in WT and *yju3*Δ cells overexpressing GFP and in *yju3*Δ cells overexpressing GFP-MGL. Cells cultivated to the late log-phase were labeled with [^14^C]palmitic acid. Lipids were separated by TLC, and the radioactivity associated with FAEE was determined. *C*, FAEE hydrolase activity of yeast 1000 × *g* supernatants. GFP-MGL was overexpressed in *yju3*Δ cells, and the cellular FAEE hydrolase activity was determined in comparison with GFP-expressing WT and *yju3*Δ cells. *D*, confocal fluorescence microscopy of AML-12 cells stably overexpressing MGL-ECFP or ECFP. LDs were detected using LipidTOX™ Deep Red. *Scale bars*: 10 μm. *E*, FAEE hydrolase activity of AML-12 cell homogenates stably overexpressing ECFP or MGL-ECFP. *F*, incorporation of [^14^C]oleic acid into FAEE in AML-12 cells stably overexpressing ECFP or MGL-ECFP. Cells were incubated with [^14^C]oleic acid in the absence or presence of ethanol and JZL-184. Subsequently, lipids were separated by TLC, and the radioactivity associated with FAEE was determined. *G*, lipid analysis of AML-12 cell homogenates and isolated LDs. Cells were incubated with oleic acid, ethanol, and JZL-184. Subsequently, lipids of cell homogenates and isolated LDs were analyzed by TLC. *H*, distribution of lipid classes between soluble/LD and membrane fractions. AML-12 cells were incubated with [^14^C]oleic acid, ethanol, and JZL-184. Subsequently, lipids were separated by TLC, and the radioactivity associated with individual lipid classes within membrane or soluble/LD fractions was determined. *PL*, phospholipids. Data are presented as the means ± S.D. (*n* = 3). Statistical significance was determined using Students unpaired *t* test. *, *p* < 0.05; *n.s.*, not significant.

Yju3p was previously identified as the yeast orthologue of mammalian MAG lipases (31). We, therefore, asked whether FAEE hydrolase activity was a general feature of this protein family. To test this possibility, we overexpressed GFP-tagged murine MGL in *yju3*Δ cells and measured cellular FAEE levels after radiolabeling. Overexpression and localization of MGL were confirmed by fluorescence microscopy. In agreement with our previous study, MGL exclusively localized to LDs in yeast (31). WT and *yju3*Δ cells expressing GFP served as controls (Fig. 6*A*). As shown in Fig. 6*B*, overexpression of MGL in *yju3*Δ cells attenuated the accumulation of FAEE and reduced cellular FAEE levels to WT. Furthermore, MGL overexpression increased cellular FAEE hydrolase activity of *yju3*Δ to WT level, indicating that MGL exhibits FAEE hydrolase activity (Fig. 6*C*). Next, we investigated the contribution of MGL to FAEE catabolism in the murine hepatocyte cell line AML-12, which forms FAEE in response to combined treatment with FAs and ethanol. To test the consequences of increased MGL levels on FAEE metabolism, we stably overexpressed an ECFP-tagged version of the enzyme using lentiviral gene transfer. Confocal fluorescence microscopy revealed that, similar as in yeast cells, MGL was closely associated with LDs in AML-12 cells (Fig. 6*D*). Overexpression of MGL-ECFP led to a 3.1-fold increase in cellular FAEE hydrolase activity *in vitro* as compared with ECFP-expressing control cell extracts (Fig. 6*E*). When exposed to ethanol, MGL-ECFP expressing cells showed a significant reduction in FAEE levels by 46% as compared with ECFP-expressing control cells (Fig. 6*F*). In contrast, treatment of AML-12 cells with the specific MGL inhibitor JZL-184 caused a 6.9-fold increase in FAEE levels in comparison to control cells (52). JZL-184 treatment also fully reversed the effect of MGL-ECFP expression on cellular FAEE levels, resulting in a similar accumulation of FAEE in JZL-184-treated MGL-ECFP- and ECFP-expressing cells (Fig. 6*F*). Taken together, these data suggest that MGL acts as a FAEE hydrolase in mammalian cells and regulates cellular FAEE levels in response to ethanol challenge. Because MGL was found to localize to LDs, we finally asked if FAEE are deposited in LDs like other hydrophobic esters such as TAG and SE. To test this, we induced FAEE formation in AML-12 cells and isolated LDs using ultracentrifugation. Lipid extracts of cell homogenates and isolated LDs were analyzed by TLC. As shown in Fig. 6*G*, FAEE were detected after ethanol treatment in both, cell homogenates, and isolated LDs. Radiolabeling experiments showed that >80% of the cellular FAEE formed under these conditions partitioned into the LD fraction (containing most of cellular TAG and SE), whereas ∼20% were associated with the phospholipid (*PL*)-containing membrane fraction (Fig. 6*H*). This finding suggests that FAEE are indeed deposited in neutral lipid stores and re-mobilized by LD-associated MGL.

## Discussion

FAEE metabolism, its regulation, and role in cellular physiology are poorly understood. In this study we identified and characterized Yju3p as a major cellular FAEE hydrolase in yeast. Yju3p deficiency resulted in a 90% decrease in the cellular FAEE hydrolase activity and a concomitant increase in cellular FAEE levels, demonstrating a major role for Yju3p in FAEE catabolism. Yju3p was previously identified as the principal MAG lipase in yeast and a functional orthologue of mammalian MGL (31). Here we identified a novel function for Yju3p as a major FAEE hydrolase and provide evidence for a dual function of this enzyme in ethanol and glycerolipid metabolism.

In *S. cerevisiae*, FAEE are a natural byproduct of ethanol fermentation. To date, the biological significance of FAEE formation in yeast has not been fully elucidated. It was suggested that FAEE function as chemoattractants that promote yeast dissemination (27, 53). From a biotechnological point of view, FAEE contribute to the aroma of alcoholic beverages (27) and may be utilized as a natural source of biodiesel. Thus, manipulating metabolic pathways that affect FAEE levels is of significant industrial interest, and deletion of FAEE hydrolases such as Yju3p may serve as a tool to increase FAEE yield. Of note, our data suggest that loss of Yju3p neither affected cell growth on fermentable or non-fermentable carbon sources nor cellular ethanol tolerance; this rather excludes a role for FAEE formation as a mechanism for EtOH detoxification in yeast.

In humans, FAEE are non-oxidative metabolites of ethanol that accumulate in tissues after ethanol consumption. FAEE are detected in blood and hair samples of social drinkers and alcoholics and are used as biomarkers for ethanol intake (54, 55). High levels of FAEE were shown to elicit toxic effects on cultured cells and tissues and may contribute to tissue damage upon excessive alcohol abuse (12, 13, 15, 16, 56). Several lines of evidence demonstrate that MGL plays a major role as FAEE hydrolase in mammalian cells. First, expression of MGL in yeast mutants lacking the orthologue Yju3p reversed FAEE accumulation to WT levels; second, under these conditions cellular FAEE hydrolase activity was significantly increased *in vitro*, and third, pharmacological inhibition of MGL in AML-12 cells drastically exacerbated FAEE formation. Together, these data support the concept that MGL and its yeast orthologue represent evolutionarily conserved FAEE hydrolases that counteract the formation of FAEE in eukaryotic cells upon ethanol exposure.

To date, little is known about the intracellular sites of FAEE formation and catabolism. Several biochemical studies demonstrated the presence of FAEE synthase, AEAT, and FAEE hydrolase activities in microsomal membranes (17, 19, 20, 26). The yeast AEAT (Eht1p) was previously shown to localize to LDs (47, 48). We detected the highest specific FAEE hydrolase activity in the LD fraction, suggesting that LDs may represent a major site of LD synthesis and degradation. Consistent with our study, the localization of both Yju3p and MGL at LDs is supported by a wealth of proteomic studies that identified MGL and Yju3p in the proteome of purified LDs from different tissue sources as well as from yeast (46–48, 57–60).

**FIGURE 7.**
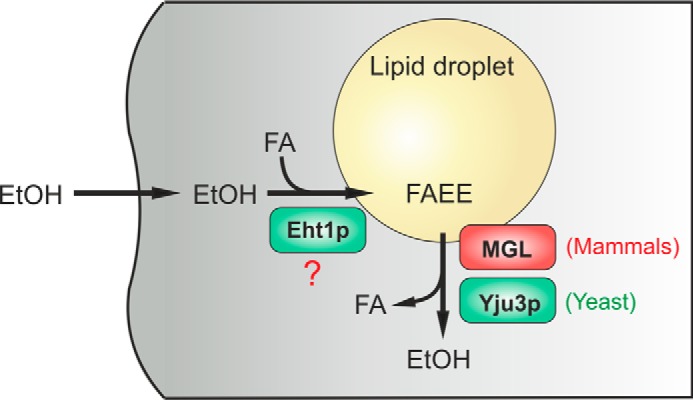
**An evolutionarily conserved family of LD-associated hydrolases controls FAEE metabolism.** In eukaryotic cells ethanol is esterified with FA to form FAEE by FAEE synthases and AEATs (*e.g.* Eht1p). FAEEs are sequestered in the hydrophobic core of LDs. Yju3p and MGL represent an evolutionarily conserved class of LD-resident enzymes, which hydrolyze FAEE to ethanol and FA in yeast and mammalian cells, respectively. We propose that LDs provide a protective environment for potentially toxic FAEE and enable their controlled remobilization by LD-resident hydrolases.

Intracellular LDs consist of a neutral lipid core containing mainly TAG and SE that are surrounded by a phospholipid monolayer. However, their composition strongly varies depending on the cell type and may also include other hydrophobic esters such as retinyl esters or neutral ether lipids (61). It is, therefore, conceivable that, similar to other hydrophobic esters, FAEE are sequestered in LDs. In support of this concept, we found that FAEE co-fractionated with LDs of AML-12 cells together with TAG and SE. Interestingly, excessive LD formation is a hallmark of alcoholic fatty liver disease, a condition that predisposes to steatohepatitis and liver cirrhosis (4). Thus, ethanol exposure may directly affect the composition of LDs via the formation of FAEE. The hydrophobic core of LDs may, therefore, provide a suitable environment to transiently store toxic FAEE and may facilitate a controlled metabolism of FAEE to prevent their potentially harmful interaction with other cellular organelles (summarized in Fig. 7). In line with this concept, LDs have been previously shown to serve as a transient buffer for potentially toxic hydrophobic compounds such as squalene or FAs (62, 63).

Until now, mammalian MGL has been implicated mainly in two physiological settings: fat mobilization and lipid signaling. In adipose tissue, MGL catalyzes the final step in an enzymatic cascade termed lipolysis, which ultimately leads to the mobilization of glycerol and FA from cellular TAG stores (64, 65). The second known function of MGL concerns its ability to hydrolyze the endocannabinoid 2-arachidonoylglycerol, a natural ligand of cannabinoid receptors (66). Hydrolysis of endocannabinoid 2-arachidonoylglycerol (2-AG) by MGL limits endocannabinoid 2-arachidonoylglycerol action at cannabinoid receptors and creates a pool of arachidonic acid, which can be further metabolized to eicosanoids (52, 68, 69). Here we report a third function of MGL as FAEE hydrolase. MGL degrades FAEE transiently stored in LDs and may also counteract the formation of these potentially toxic byproducts of ethanol metabolism in other organelles. Interestingly, postnatal ethanol exposure of mice was recently shown to increase MGL levels in brain (67). Whether this up-regulation of MGL represents an adaptive response to counteract FAEE formation or, rather, a regulatory response of the endocannabinoid system is currently unknown. Clearly, *in vivo* studies are required to further elucidate the role of MGL in ethanol metabolism and toxicity in mammals. Taken together, our findings suggest a functional versatility of MGL family members in glycerolipid and ethanol metabolism that is evolutionarily conserved from yeast to mammals.

## Author Contributions

C. H. and R. Zimmermann conceived and coordinated the study. C. H.., S. D. K., and R. Zimmermann wrote the paper. C. H.., M. R., P. A., S. G., H. W., and U. T. performed the experiments. T. O. E. performed lipid analyses. S. D. K., M. O., and R. Zechner provided intellectual support and supervision. All authors reviewed the results and approved the final version of the manuscript.

## Footnotes

3 The abbreviations used are:

FAEE: fatty acid (FA) ethyl ester
AEAT: acyl-CoA ethanol *O*-acyltransferase(s)
MAG: monoacylglycerol
MGL: monoglyceride lipase
LD: lipid droplet
TAG: triacylglycerol
SE: steryl ester
YPD: yeast extract/peptone/dextrose
MM: minimal medium
LD: lipid droplet
Bistris propane: 1,3-bis[tris(hydroxymethyl)methylamino]propane
YPE: yeast extract/peptone/ethanol
RFP: red fluorescent protein
ECFP: enhanced cyan fluorescent protein.
